# Detection of Airway Anomalies in Pediatric Patients with Cardiovascular Anomalies with Low Dose Prospective ECG-Gated Dual-Source CT

**DOI:** 10.1371/journal.pone.0082826

**Published:** 2013-12-06

**Authors:** Hui Jiao, Zhuodong Xu, Lebin Wu, Zhaoping Cheng, Xiaopeng Ji, Hai Zhong, Chen Meng

**Affiliations:** 1 Shandong Medical Imaging Research Institute, Shandong University, Ji’nan, Shandong, People’s Republic of China; 2 Shandong Medical Imaging Research Institute, Ji’nan, Shandong, People’s Republic of China; 3 Department of Radiology, the Second Hospital of Shandong University, Ji’nan, Shandong, China; 4 Shandong University Qilu children hospital, Ji’nan, Shandong, China; University of Groningen, Netherlands

## Abstract

**Objectives:**

To assess the feasibility of low-dose prospective ECG-gated dual-source CT (DSCT) in detecting airway anomalies in pediatric patients with cardiovascular anomalies compared with flexible tracheobronchoscopy (FTB).

**Methods:**

33 pediatrics with respiratory symptoms who had been revealed cardiovascular anomalies by transthoracic echocardiography underwent FTB and contrast material–enhanced prospective ECG-triggering CT were enrolled. The study was approved by our institution review board and written informed consent was obtained from all patients’ guardian. DSCT examinations were performed to detect cardiovascular abnormalities using weight-adjusted low–dose protocol. Two radiologists independently performed CT image analysis. The FTB reports were reviewed by an experienced pulmonologist. The sensitivity, specificity, positive predictive value (PPV), negative predictive value (NPV), and accuracy of DSCT in the detection of airway anomalies were assessed. The tracheobronchial stenoses revealed on FTB were graded. Effective radiation dose was calculated.

**Results:**

Thirty cases were diagnosed with tracheobronchial narrowing and/or abnormality in 33 patients by FTB, while 3 patients had normal FTB findings. Twenty-eight cases were diagnosed with airway anomalies by CT, of which 27 were correct positive. 3 patients with normal findings at CT had findings of tracheobronchial narrowing due to tracheobronchomalacia at inspiration at FTB. Sensitivity and specificity of CT were 90.0% (95% CI: 72.3%, 97.4%) and 66.7% (95% CI: 12.5 %, 98.2 %), respectively. PPV and NPV were 96.4% (95% CI: 79.8 %, 99.8%) and 40.0% (95% CI: 7.3%, 83.0%), respectively. Overall accuracy of DSCT in detecting airway anomalies in pediatrics with cardiovascular anomalies was 87.9% (95% CI: 74.5%, 97.6%). In grading of tracheobronchial stenosis, images from CT correlated closely (*r* = 0.89) with those of FTB. Mean effective dose was 0.60±0.20 mSv.

**Conclusion:**

In pediatric patients, ECG-triggered CT to evaluate congenital cardiovascular anomalies can also be used to diagnose and characterize fixed airway involvement in relation to the vascular structures.

## Introduction

Respiratory symptoms are often present in infants and children with congenital cardiac anomalies. These symptoms mostly result from large left to right shunt, flow obstruction of the systemic ventricle failure, and the rare condition of vascular airway compression [1]. In addition, absent pulmonary valve syndrome and intrinsic pathology also contribute to airway obstruction [2].Airway anomalies in infants and children associated with congenital heart disease, such as the occurrence of vascular slings or rings, may complicate the natural and surgical history of the patients and significantly increase mortality and morbidity [3-8]. 

Multi-detector computed tomography (MDCT) with improved spatial and temporal resolution, larger anatomic coverage and high quality reconstructions has become an important examination tool in the detection of the tracheobronchial anomalies and stenoses [9,10]. It was reported that prospective ECG triggering dual-source computed tomography (DSCT) angiography with a very low effective radiation dose allowed the accurate diagnosis of complex congenital heart disease in infants and children [11].To our knowledge, there have been no studies in the detection of airway anomalies in infants and children with cardiovascular anomalies using prospective ECG triggering DSCT. This study aims to evaluate the feasibility of low-dose DSCT in detecting suspected airway anomalies in the pediatric patients with cardiovascular anomalies using flexible tracheobronchoscopy (FTB) as the reference standard. 

## Materials and Methods

### Patients

The study was approved by the Shandong Medical Imaging Research Institute ethics board with written informed consent obtained from all patients’ guardian. Infants and children who had undergone transthoracic echocardiography which revealed the presence of cardiovascular anomalies and who had undergone FTB for possible tracheobronchial narrowing and/or anomaly were enrolled in the study and underwent DSCT for detection of cardiovascular abnormalities between August 2010 and December 2012. Indication for performing FTB included: recurrent/persistent pneumonia, persistent atelectasis, and/or abnormal findings in pulmonary function tests or physical examination (abnormal breathing sounds, stridor, wheezing without response, dyspnea). Patients with known allergy to contrast material, or renal insufficiency and those whose parents dissented for FTB and/or DSCT were excluded from the study. A total of 33 patients (16/17 male/female) with a history of persistent airway obstruction (abnormal breathing sounds, persisting or recurrent pulmonary infections), and/or presented stridor with related symptoms (cyanosis, fever, hemoptysis or dyspnea at feeding) were enrolled in this study, with ages ranging from 1 month to 7.75 years (mean 1.27±1.90 years) and weight from 3.0 kg to 19.0 kg (mean 6.93±3.97 kg). 

### Fiberoptic tracheobronchoscopy (FTB)

Flexible bronchoscopy was performed by an experienced pulmonologist using a videobronchoscope (F-P260F or BF-XP260F, Olympus, Japan) with the patients under local anesthesia. CT examinations of the airways were performed after flexible bronchoscopy. The mean interval between CT examination and flexible bronchoscopy was 2.5 days (ranging from 0 to 7 days). To provide a reference standard, an experienced pulmonologist reviewed the flexible bronchoscopy reports without knowledge of the CT findings and clinical histories (derived from written bronchoscopy reports). Endobronchial photographs were obtained at stenotic and other abnormal sites. The airways were classified into six locations as follows: 1) location I, upper third of the trachea; 2) location II, middle third of the trachea; 3) location III, lower third of the trachea; 4) location IV, right main bronchus; 5) location V, left main bronchus[12]. In addition, we defined lobe bronchus as location VI. The grade of tracheobronchial narrowing was categorized semiquantitatively as grade 1 (0<luminal narrowing < one third), grade 2 (luminal narrowing **≥**one third but < two thirds), or grade 3 (luminal narrowing **≥**two thirds) [13].

### CT techniques

All patients underwent prospective ECG-triggering DSCT (Somatom Definition Flash, Siemens Healthcare, Forchheim, Germany). Patients were examined in the supine position with elevated arms. CT parameters were set as follows: 0.28 s gantry rotation time, 2×64×0.6 mm detector collimation, a slice collimation 2×128×0.6 mm by z-flying focal spot technique. The centre of the data acquisition window was set at 40% of the R-R interval. All DSCT examinations were performed by weight-adjusted low –dose protocol while patients were freely breathing. Body weight-based adjustments of tube voltage and tube current were performed: <6 kg, tube voltage 80 kV, tube current 40–59 mAs; 6–10 kg, tube voltage 80 kV, tube current 60–79 mAs; >10 kg, tube voltage 80 kV, tube current 80–120 mAs.

Sedation was achieved with oral administration of chloral hydrate (50–100 mg per kilogram body weight, maximum dose of 2000 mg) according to the patient’s body weight. In general, sedation was unnecessary in patients over 5 years old who can cooperate well. Data acquisition was performed in the cranio-caudal direction, followed with range from the thoracic inlet to the level of the diaphragm. The duration of the CT data acquisition was 5.23–9.18 s.

A dual-head power injector (Stellant; Medrad, Indianola, PA) was used. The saline flush technique was applied for all injections to reduce the artifacts caused by undiluted intravascular contrast agent. Intravenous contrast agent (Schering Ultravist, Iopromide, 350 mg I/ml, Berlin, Germany) was administered via a peripheral vein. We used 1.5 ml/kg of contrast medium followed by the saline flush with half volume of the total contrast medium. The delay time was set to 25 s. Injection rate was calculated as the total injected volume divided by 25 s.

### Image processing and analysis

All images were reconstructed with a slice thickness of 0.75 mm and an increment of 0.5 mm (collimation, 0.6 mm) with a smooth-tissue convolution kernel (B30f). All images were transferred to an external workstation (Multi Modality workplace, Siemens, Forchheim, Germany) for further analysis. In addition to conventional axial slices, multiplanar reformation (MPR), maximum intensity projection (MIP) and volume rendering (VR) were performed to visualize the cardiovascular and airway anomalies by two radiologists with more than 10 years’ experience in pediatric thorax radiology who were blinded to FTB findings and clinical histories. Both axial and MPRs images were viewed in standard soft-tissue window settings (centre, 80 HU; width, 400 HU) and in lung window settings (centre, −600 HU; width, 1400 HU). All measurements were performed in lung window settings (centre, −600 HU; width, 1400 HU) [14]. 

The airways were classified into six locations as follows: 1) location I, upper third of the trachea; 2) location II, middle third of the trachea; 3) location III, lower third of the trachea; 4) location IV, right main bronchus; 5) location V, left main bronchus[12]. Additionally, we defined lobe bronchus as location VI. Each case of bronchial stenosis was rated as grade 1 (luminal narrowing < one third), grade 2 (luminal narrowing ≥ one third but < two thirds), or grade 3 (luminal narrowing ≥ two thirds) [13]. Assessments by CT were defined as overestimations or underestimations according to whether the particular stenosis had been assigned a higher or lower grade at the review of the findings of flexible bronchoscopy. In addition, airway abnormalities (accessory bronchi, bronchial hypoplasia, and abnormal branching, et al) were documented. 

 We classified all the CT findings as tracheobronchial narrowing and abnormality, using 5-score scale to evaluate the airway at each location as follows: score 0 (absence of tracheobronchial narrowing and abnormality ), score 1 (0<luminal narrowing < one third), score 2 (luminal narrowing ≥one third but < two thirds), and score 3 (luminal narrowing ≥two thirds), score 4 (airway abnormality). For any disagreement in data analysis between the two observers, consensus agreement was achieved. 

### Radiation dose evaluation

The parameters for volume CT dose index (CTDIvol) and dose-length product (DLP) were obtained from the patient protocol that summarized the relevant radiation exposure parameters of each CT examination. The effective radiation dose (mSv) was calculated from the DLP (mGy·cm) multiplied by 2.3 to adapt it to the 16-cm phantom (the DLP for the body surface area was given for a 32-cm phantom on the scanner protocol and the conversion factor of 2.3 is scanner specific for paediatric examinations at 80 kV as provided by the manufacturer). Effective radiation dose was estimated by multiplying the corrected DLP value by a organ-specific conversion coefficient ED (mSv) =DLP*γ (γ: organ-specific conversion coefficient) [15]. Infant-specific dose-length product conversion coefficients were given for a 16-cm phantom: a coefficient of 0.039 mSv/[mGy·cm] for patients under 4 months, 0.026 mSv/[mGy·cm] between 4 months and 1 year of age, and 0.018 mSv/[mGy·cm] between 1 year and 6 years of age [16].

### Statistical Analysis

A positive finding was defined as tracheobronchial narrowing and/or an airway abnormality noted at either FTB or CT, whereas a negative finding was defined as absence of airway stenosis and airway abnormality at either FTB or CT. FTB results were taken as the reference standard. The sensitivity, specificity, positive predictive value (PPV), negative predictive value (NPV) and accuracy of CT were calculated from 2X2 contingency tables, with calculation of the 95% confidence intervals being calculated.

Spearman’s rank order correlation (r) was calculated to measure the strength of correlation between the results of CT and flexible bronchoscopy for grading of tracheobronchial stenosis. A p value of less than 0.05 was considered significant.

Interobserver agreement for evaluation of tracheobronchial narrowing and abnormality was assessed by kappa test, and к-values of 0.80-1.00 were considered to indicate good agreement.

## Results

### FTB and CT findings

In 30 (91%) of 33 patients, tracheobronchial narrowing and/or abnormality was present at FTB, while 3 patients had normal FTB results. 

Twenty-eight cases were diagnosed with tracheobronchial narrowing and/or abnormality in 33 patients by CT. 27 patients were confirmed by FTB, while 1 patient with diagnosis of tracheobronchial narrowing on CT was diagnosed with mucus by FTB. In the 5 patients with negative CT results, 2 patients were also negative at FTB, while 3 patients were diagnosed with tracheobronchial narrowing due to tracheobronchomalacia by FTB (Table 1). 

**Table 1:** Comparison of results: fiberoptic tracheobronchoscopy (FTB) and dual-source computed tomography (DSCT).

DSCT	FTB		Total
	Positive	Negative	
Positive	27	1	28
Negative	3	2	5
Total	30	3	33

For all 27 patients with airway anomalies detected by both DSCT and FTB, 26 sites of tracheobronchial stenoses were detected. The described location was consistent with each other, including: location I (4 sites), location II(1 sites), location III(5 sites), location IV(4 sites), location V(10 sites), location VI(2 sites) . In 26 stenoses, grading degree of 23 stenoses detected by both CT and FTB was consistent with each other, including: grade 1 (5 stenoses), grade 2(11 stenoses), grade 3 (7 stenoses). Based on CT, 1 stenosis was overestimated by 1 grade, and 2 stenoses were underestimated by 1 grade.

**Figure 1:**
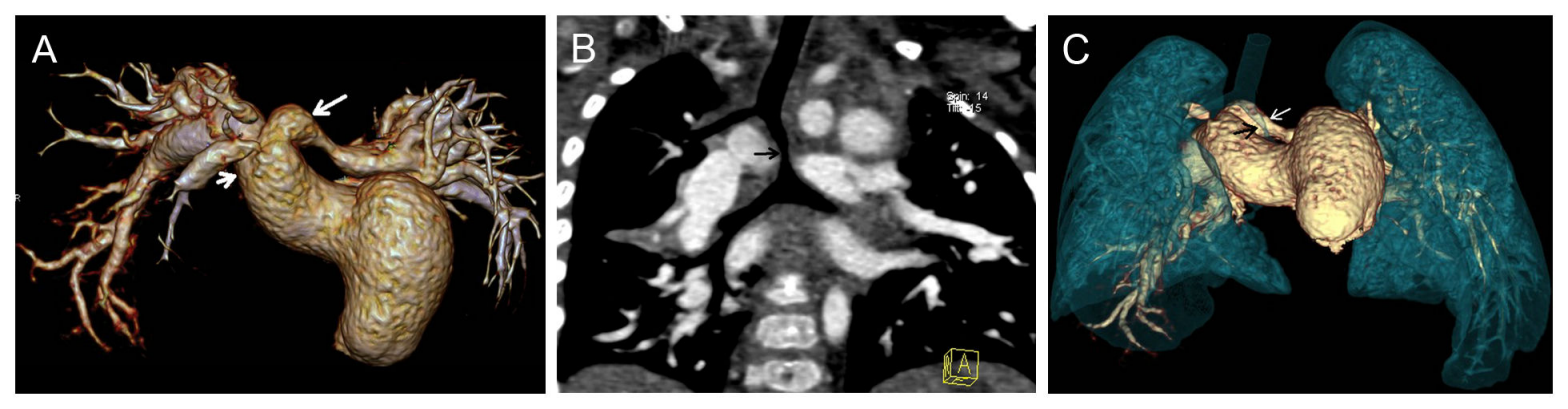
A 1-month girl with congenital heart disease (pulmonary artery sling and ventricular septal defect) was demonstrated the presence of bridge bronchus and bronchial narrowing. A. Volume Rendering (VR) showed the thickening of the pulmonary trunk and the left pulmonary artery (short white arrow) arose from the proximal right pulmonary artery (long white arrow). B. CT coronal image showed the bridge bronchus and bronchial stenosis (black arrow) caused by the anomalous origin of the left pulmonary artery. C. Volume Rendering (VR) of the tracheobronchial tree and the thoracic vascular demonstrated the bridge bronchus and bronchial stenosis (black arrow) due to anomalous origin of the left pulmonary artery (white arrow) compression.

All patients with proven airway abnormalities without narrowing (n =10) had correctly identified findings at CT, including bridge bronchus (n=3) (Figure 1B), tracheal bronchus (n=4) and segmental bronchial agenesis (n=3) (Table 2). 

**Table 2:** Summary of CT findings of airway anomalies in 33 pediatric patients with cardiovascular anomalies.

Type of airway anomalies demonstrated by CT	Cardiovascular abnormalities demonstrated by CT	Age (year)
segmental bronchial agenesis	Scimitar syndrome (2 cases)	0.25~5.8
	Anomalous origin of the brachiocephalic trunk (1 case)	0.17
bridge bronchus	PAS+ASD (1 case)	0.17
	PAS+RAA+aberrant left subclavian artery (1 case)	2
	ASD+PH (1 case)	0.42
	PAS (1 case)	0.25
	PAS+PH+ASD (1 case)	0.17
	PAS+VSD+PH (1 case)	0.08
tracheal bronchus	PH+persistent left superior vena cava (1 case)	0.42
	PAS (1 case)	1
	Tetralogy of Fallot (1 case)	0.08
	PDA+PH+ASD (1 case)	0.25
tracheobronchial stenosis	right pulmonary artery and vein hypoplasia+ASD (2 cases)	0.17~0.25
	PAS (5 cases)	0.08~3
	DAA (1 case)	1.75
	PH (2 case)	0.58~2.2
	PAS +VSD+PH (1 case)	0.75
	PDA+PH (3 cases)	0.25~0.42
	Tetralogy of Fallot+RAA (1 case)	3
	Coarctation of the aorta+VSD+ASD+PH (1case)	0.08
	VSD+PH (1 case)	0.83
	PAS+RAA (1 case)	2
	PH+persistent left superior vena cava (1 case)	0.42
	VSD+PH (1 case)	0.08

PAS=pulmonary artery slingASD=atrial septal defectRAA= right aortic arch PH= pulmonary hypertensionVSD= ventricular septal defect PDA= patent ductus arteriosusDAA= double aortic arch

**Figure 2:**
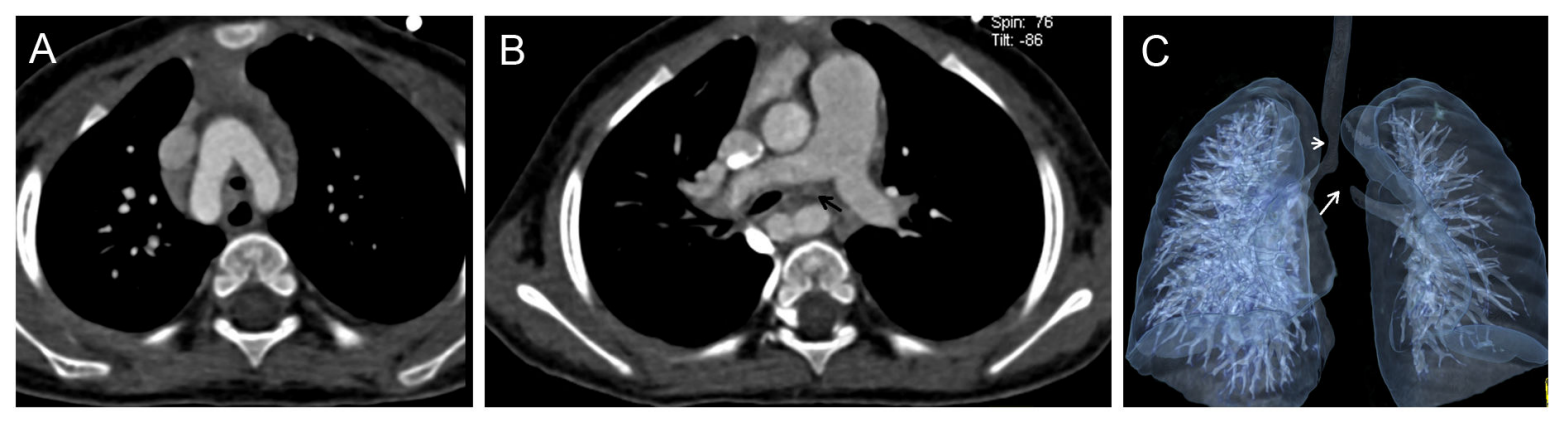
A 21-month boy with double aortic arch and subsequent tracheobronchial narrowing. In addition, a nonvascular left main bronchus stenosis was detected. A. CT axial image demonstrated double aortic arch which caused encirclement of the trachea. B. CT axial image showed the soft issue lesion compressed the left main bronchus, and local lumen was narrow (black arrow). C. Volume Rendering (VR) show the one tracheal stenosisi (short white arrow) caused by double aortic arch and the other severe bronchial narrowing (long white arrow) due to a nonvascular compression.

**Figure 3:**
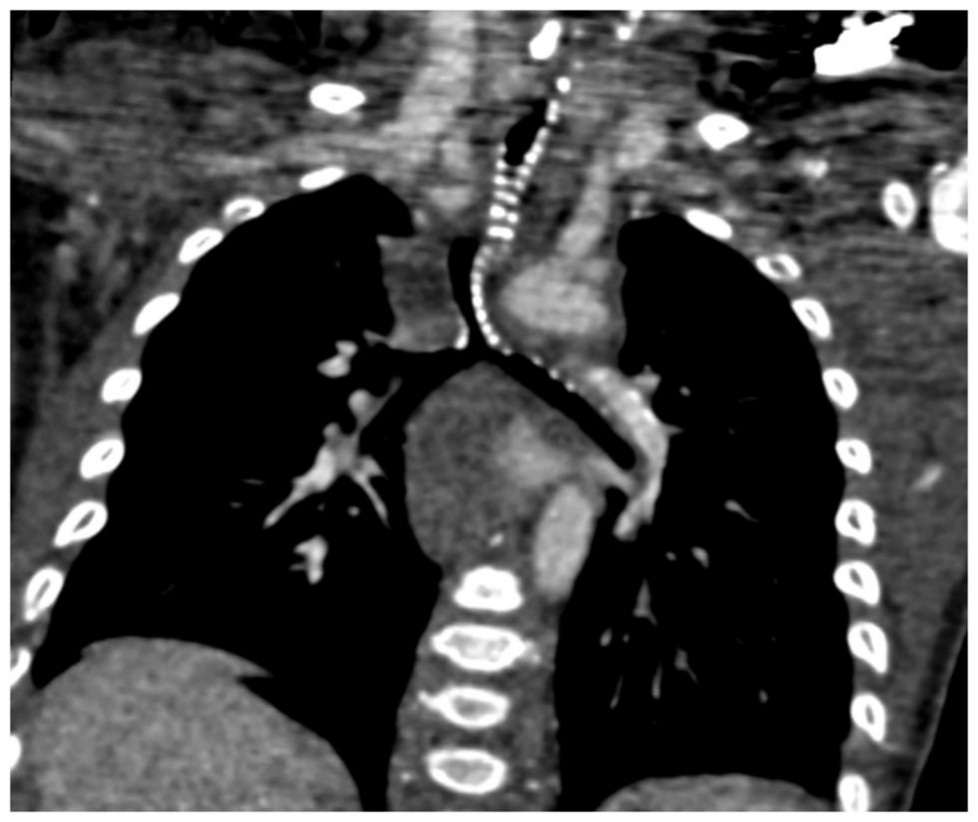
A 7-month old infant with pulmonary hypertension was demonstrated tracheobronchial widespread stenoses at CT and the fiberscope could not pass the lesion. CT coronal image demonstrated the widespread ossification of tracheobronchial cartilage and irregular stenoses.

**Figure 4:**
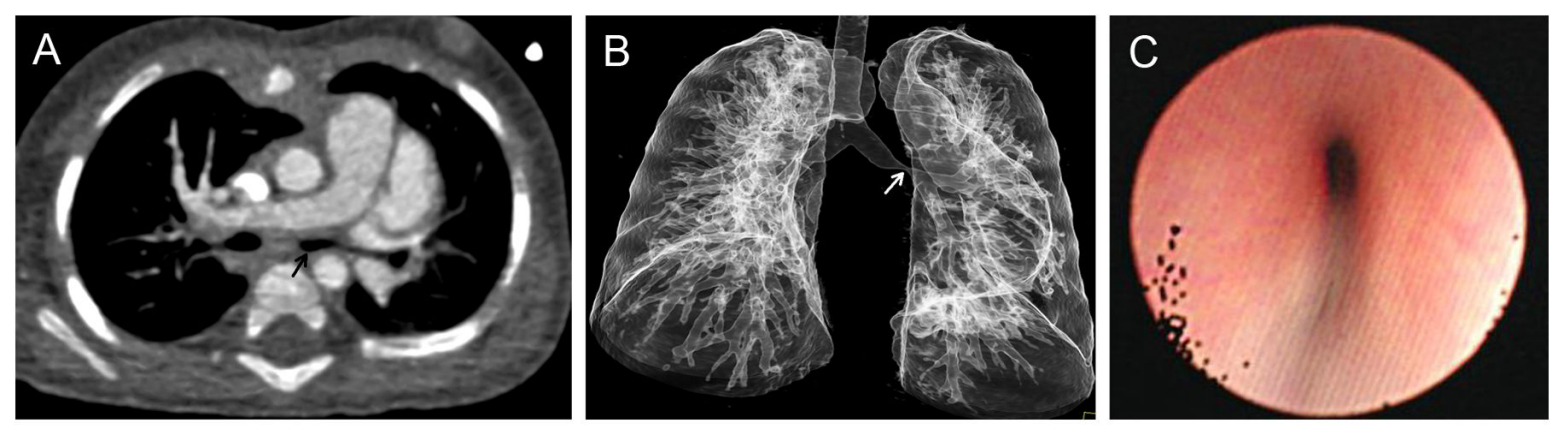
A 6-month girl with ventricular septal defect was detected by both CT and FTB with the left main bronchus compression and the local lumen became stenotic. A. CT axial image demonstrated the left main bronchus stenosis due to surrounded compression by anonymous soft tissue (black arrow). B. Volume Rendering (VR) of the tracheobronchial tree showed the local stenosis (white arrow) at the left main bronchus. C. FTB confirmed the left main bronchus stenosis, which noted extralumen pulsatile compression of the bronchus.

Vascular abnormalities that caused airway narrowing were confirmed in 9 (45%) of 20 patients with airway stenosis demonstrated by FTB. Pulmonary artery sling (PAS) was the most common finding (n=5) (Figure 1), while PAS was associated with bridge bronchus in 3 cases. The other vascular abnormalities that caused airway narrowing included right aortic arch (n=2), aberrant left subclavian artery (n=1) and double aortic arch (n=1) (Figure 2). Nonvascular airway stenosis was detected in 11 (55%) of 20 patients. CT revealed a 7-month old infant to have tracheobronchial stenosis due to widespread ossification of tracheobronchial cartilage while the fiberscope could not pass the lesion (Figure 3). 3 patients presented trachea stenosis due to anonymous soft tissue compression (Figure 4). FTB demonstrated 4 cases with tracheobronchial narrowing due to bronchial wall fibrosis. 3 cases with airway stenosis due to tracheomalacia were confirmed at FTB (Table 3).

**Table 3:** Vascular and nonvascular abnormalities that cause airway narrowing.

vascular compression	nonvascular airway stenoses
pulmonary artery sling (5cases)	ossification of tracheobronchial cartilage (1 case)
double aortic arch (1 case)	local bronchial wall fibrosis (4 cases)
right aortic arch (2 cases)	anonymous soft tissue compression (3cases)
aberrant left subclavian artery (1 case)	bronchomalacia (3cases)

### Statistical Parameters

Sensitivity and specificity of CT were calculated as 90.0% (95% CI: 72.3%, 97.4%) and 66.7% (95% CI: 12.5 %, 98.2 %) respectively, while PPV and NPV were 96.4% (95% CI: 79.8 %, 99.8%) and 40.0% (95% CI: 7.3%, 83.0%) respectively. Overall accuracy was 87.9% (95% CI: 74.5 %, 97.6%).

The correlation between the findings of CT and FTB for grading of tracheobronchial stenosis was excellent (*r* = 0.89). 

There was good agreement (к=0.81) for evaluation of tracheobronchial narrowing and abnormality between the two reviewers.

### Radiation Dose

The mean volume CT dose index was 0.87±0.34 mGy (range: 0.61–1.98).The mean dose-length product was 10.15±5.39 mGy·cm (range: 5–34) resulting in an estimated mean effective radiation dose of 0.60±0.20 mSv (range: 0.33–1.41). 

## Discussion

Congenital tracheobronchial variant or anomaly has been reported with an incidence of 0.1% to 2% in population [17]. In addition, the incidence of bronchial dysplasia in children with congenital heart disease (CHD) associated with ventricular septal defect was found to be higher than those without ventricular septal defect (33.7% vs 15.0%), which suggested the intimate relationship between bronchial dysplasia and congenital heart disease [18]. 

 Complete or partial vascular rings, external compression by anomalous cardiovascular anatomy, and tracheomalacia are the most common causes of tracheal obstruction. The term ‘vascular rings’ encompasses a wide range of aortic arch abnormalities that cause encirclement of the trachea and esophagus. Complications can be divided into 2 groups: direct vascular compression leading to airway compromise and swallowing difficulties, and secondary bronchomalacia and stenoses resulting from prolonged compression and degeneration of previously normal cartilage [19]. In the present study, congenital vascular abnormalities accounted for a substantial number of fixed tracheobronchial stenoses in children. MDCT was indispensable to confirm the diagnosis of cardiovascular lesion, coexistent airway pathology and their relationship, especially in those with a high suspicion of vascular airway compression.

 Fiberoptic tracheobronchoscopy (FTB) serves as the gold standard in patients with stridor and respiratory distress. FTB allows direct visualization of the airway lumen, biopsy sampling and foreign body removal, and has a diagnostic and therapeutic role by assessing the airway both anatomically and dynamically [13,20]. It has the disadvantage of being an invasive procedure that can cause patients’ suffering. Also, the fiberscope can not pass through high-grade stenosis to evaluate the poststenotic airway segments, and cannot provide information outside the airway lumen [13,21]. MDCT with three-dimensional imaging is useful in supplying information about severe tracheobronchial stenoses and depicts the poststenotic airway segments. High temporal and spatial resolution with high quality reconstructions enables CT to provide the depiction of the tracheo-bronchial tree, pulmonary parenchymal assessment, as well as evaluation of the mediastinal vascular compression leading to airway compromise. 

In our study, we used a low dose CT protocol with tube current reduction and voltage adjustment according to patients’ weight. Collimation was chosen as submillimeter collimation (0.6 mm). A collimation of less than 1 mm is particularly needed in pediatric patients to acquire thin-section images. A previous study on the value of thoracic multidetector CT in pediatric patients reported a mean effective dose of 1.1 mSv (range, 0.5–1.8 mSv) [9]. Our results indicated that by using a low-dose protocol, substantial reduction of radiation exposure to a mean effective dose of 0.60±0.20 mSv (range: 0.33–1.41) could be achieved. Prospective ECG-triggering DSCT could depict pediatric tracheobronchial anomalies with a sensitivity of 90.0%, a PPV of 96.4%, and an overall accuracy of 87.9%.

There were 3 false-negative findings based on DSCT (patients with tracheobronchomalacia and normal findings at CT). This could be explained by the fact that tracheomalacia and bronchomalacia do not cause a fixed narrowing. CT just shows a snapshot of the respiratory cycle, hence we missed three stenoses. A possible way to solve this would be a biphasic CT examination in inspiration and expiration; however this would lead to an increased radiation exposure. Moreover, pediatric patients were unable to cooperate well regarding the breath holding. There was 1 false-positive finding with DSCT (mucus which was interpreted as an airway stenosis on CT was noted on FTB, and was removed through irrigation and suction). Retained secretions and artifacts may result in false-positive findings in CT. Also, CT cannot show the morphology, vascularity, or color of the mucosa [22].

Misinterpretation of stenosis with CT may be explained by the fact that CT reviewers marked the degree of stenosis during inspiration, while the minimal cross-sectional area at the end of expiration was used on FTB. It was proposed to rate the highest degree of stenosis during normal breathing, which in most cases is at the end of expiration [12]. In addition, pediatric patients may not be able to hold their breath during CT examination, causing breathing and motion artifacts that may lead to misinterpretation of the tracheobronchial lumen [23].

We acknowledge the following limitations of our study. First, the small sample of the enrolled patients, because it is limited by the rare syndrome of, vascular induced airway compressions in children. Future studies with larger patient populations on the diagnostic accuracy of low-dose prospective ECG-triggering DSCT in airway anomalies in children with cardiovascular anomalies are needed. Second, dynamic imaging is not available. This is especially valuable for diagnosis of tracheomalacia in pediatric patients, in which the dynamic collapse of the trachea during expiration may lead to airway obstruction with development of wheeze, cough, stridor, dyspnoea, cyanosis and recurrent respiratory infections. 

## Conclusion

In children with congenital heart disease and a high suspicion of airway involvement the probability of fixed airway involvement seems high. In pediatric patients, ECG-triggered CT to evaluate congenital cardiovascular anomalies can also be used to diagnose and characterize fixed airway involvement in relation to the vascular structures. If no fixed airway abnormalities are seen, a dynamic CT or bronchoscopy may identify dynamic airway narrowing. 
